# Hypereosinophilia and Left Ventricular Thrombus: A Case Report and Literature Review

**DOI:** 10.7759/cureus.61674

**Published:** 2024-06-04

**Authors:** Aleksan Khachatryan, Hakob Harutyunyan, Mitchell Psotka, Ashot Batikyan, Tufan Cinar, Michael Khorsandi, Joel Alejandro, Vahagn Tamazyan, Margarita Sargsyan

**Affiliations:** 1 Department of Internal Medicine, University of Maryland Medical Center, Midtown Campus, Baltimore, USA; 2 Department of Internal Medicine, Maimonides Medical Center, New York, USA; 3 Department of Advanced Heart Failure and Transplant Cardiology, Inova Fairfax Medical Campus, Falls Church, USA; 4 Department of Internal Medicine, North Central Bronx Hospital, New York, USA; 5 Department of Cardiology, Johns Hopkins University, Baltimore, USA; 6 Department of Cardiology, "Heratsi" Hospital Complex № 1, Yerevan, ARM

**Keywords:** loeffler endomyocarditis, cardiac magnetic resonance imaging (cmri), absolute eosinophil count, arterial thromboembolism, restrictive cardiomyopathy, endomyocardial biopsy, eosinophilic myocarditis, left ventricular thrombosis, hypereosinophilia syndrome, hypereosinophilia

## Abstract

Left ventricular thrombus (LVT) has historically been reported as a complication of acute left ventricular (LV) myocardial infarction. It is most commonly observed in cases of LV systolic dysfunction attributed to ischemic or nonischemic etiologies. Conversely, the occurrence of LVT in normal LV systolic function is an exceptionally rare presentation and is predominantly associated with conditions such as hypereosinophilic syndrome (HES), cardiac amyloidosis, left ventricular noncompaction, hypertrophic cardiomyopathy (HCM), hypercoagulability states, immune-mediated disorders, and malignancies. Notably, hypereosinophilia (HE) has been linked with thrombotic events. Intracardiac thrombus is a well-known complication of eosinophilic myocarditis (EM) or Loeffler endomyocarditis, both of which are considered clinical manifestations of HES. We present a case of a 63-year-old male with normal LV systolic function, HE, and noncontributory hypercoagulability workup, who presented with thromboembolic complications arising from LVT. Interestingly, the diagnostic evaluation for EM and Loeffler endocarditis was nonconfirmatory. Additionally, we performed a literature review to delineate all similar cases. This article also outlines the pathophysiology, diagnosis, and treatment approaches for hypereosinophilic cardiac involvement with a specific focus on LVT.

## Introduction

Hypereosinophilia (HE) is characterized by an elevation of the absolute eosinophil count (AEC) of >1.5 × 10⁹/L (or >1,500 cells/μL) in two measurements taken at least one month apart and/or the confirmation of increased eosinophils on pathology examination. The main HE categories are primary, secondary, and idiopathic HE and hypereosinophilic syndrome (HES). The primary or clonal causes of HE are attributed to bone marrow disorders, including platelet-derived growth factor receptor alpha/beta (PDGFRA/B) rearrangements, whereas the secondary etiologies of HE include allergy/atopy, drug reactions, infections particularly parasitic etiologies, and collagen-vascular diseases [1]. HES and idiopathic HE are considered diagnoses of exclusion, and the key distinction between these two entities is the eosinophilic end-organ damage observed in HES but absent in idiopathic HE [1]. The cardiovascular manifestations of HE are variable, ranging from clinically silent cases to eosinophilic myocarditis (EM) or chronic restrictive cardiomyopathy (also known as Loeffler cardiomyopathy or endomyocarditis) with associated complications. Thrombotic presentations are one of the unfavorable complications of HES, described as venous, arterial, or intracardiac thrombi, and may be observed in as many as 21% of cases [2,3]. Intraventricular thrombi in HES are extensively described in the context of either EM or Loeffler endocarditis, which are typical cardiac manifestations of HES. However, the reports of HES causing intracardiac thrombosis beyond these two entities are limited.

The case we present serves as an example of HES potentially contributing to left ventricular thrombus (LVT) formation in the absence of typical cardiac manifestations of HES, highlighting the association between intracavitary thrombi and HES. Additionally, an extensive literature search was conducted to categorize all similar cases described in this review to enhance awareness about the association of HES with LVT, in the absence of typical cardiac involvement in HES. We systematically searched PubMed, Google Scholar, and other sources for cases with similar presentations and intracardiac thrombus published until November 2023. We used search phrases such as "hypereosinophilia and intracardiac thrombosis" and "hypereosinophilia and arterial thromboembolism" to identify all relevant reports. To minimize possible confounders, all the cases with potential alternative explanations of intracardiac thrombi, such as heart failure with reduced ejection function, the presence of autoimmune disorders, and malignancies, were excluded. Only cases that provided sufficient information about the workup, including cardiac magnetic resonance imaging (CMR) and/or endomyocardial biopsy (EMB), with no conclusive evidence of eosinophilic cardiac involvement, were considered. As a result, we identified two cases that met our search criteria.

Additionally, this review aims to provide a summary of the pathogenesis, diagnosis, and treatment of hypereosinophilic cardiac involvement based on the latest research with an emphasis on LVT.

## Case presentation

A 63-year-old male with a past medical history of hyperlipidemia, type 2 diabetes mellitus, congestive heart failure (restrictive cardiomyopathy), atrial fibrillation, HE, LVT, ischemic stroke, bilateral iliofemoral and aortic embolectomy, pulmonary hypertension (PHTN), peripheral neuropathy, hypothyroidism, and attention deficit hyperactivity disorder (ADHD) presented complaining of fatigue and shortness of breath.

The weakness had been progressing for the past month to the point that he was unable to perform basic activities at home. The shortness of breath started one day before the presentation. Additional symptoms included diminished appetite and a 50 lb weight loss over the past year. He denied chest pain, dizziness, palpitations, leg swelling, paroxysmal nocturnal dyspnea (PND), orthopnea, cough, recent infections, fever, abdominal pain, diarrhea, constipation, nausea, or vomiting.

The patient's medical history started around 15 months ago when he was diagnosed with paroxysmal atrial fibrillation. A transthoracic echocardiogram (TTE) was negative for any intracardiac thrombus at that time. He was prescribed apixaban for atrial fibrillation.

**Figure 1:**
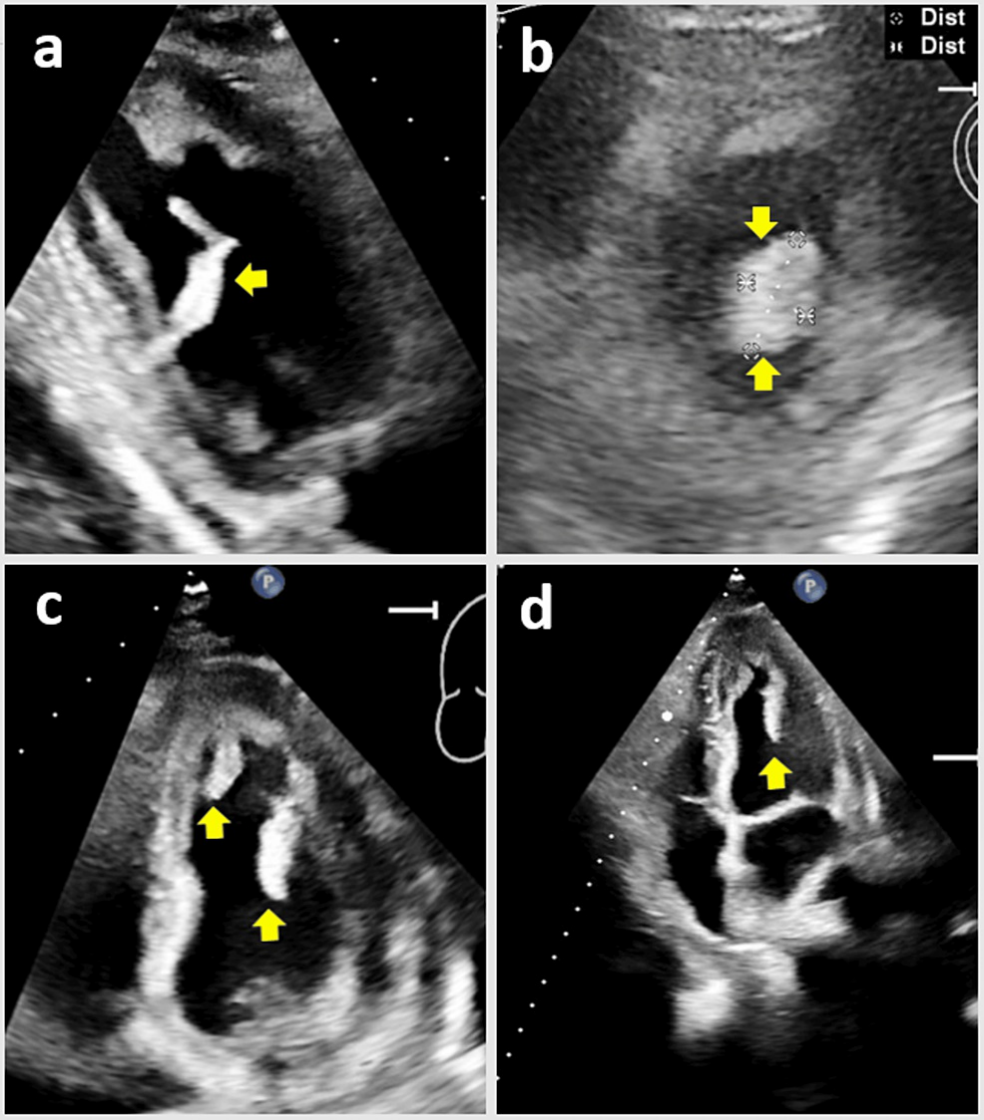
The TTE nine months ago demonstrated intraventricular thrombi when the patient presented with multiple thromboembolic complications (a and b) LV inferolateral wall thrombus on parasternal long and short axis views, respectively. (c and d) Apical and inferolateral floating thrombi on apical four-chamber view TTE, transthoracic echocardiogram; LV, left ventricular

Approximately nine months ago, the patient presented with a constellation of cardioembolic complications attributed to LVT, including stroke, left subclavian artery occlusion, infrarenal aortic occlusion with acute limb ischemia requiring thrombectomies of the distal aorta, bilateral common iliac and lower extremity arteries with bilateral iliofemoral bypass, and compartment fasciotomies. The TTE performed at that time identified normal left ventricular (LV) global and regional contractility with an ejection fraction (EF) of 65%-70%, mild mitral regurgitation (MR), and two large mobile thrombi on the apex and inferolateral walls measuring 2.3 × 0.7 cm and 2.4 × 1.0 cm, respectively (Figure 1).

That hospitalization was also remarkable for an elevated AEC of 2.47 × 10³/µL (reference range: 0.00-0.44 × 10³/µL), which was the highest recorded AEC of the patient's history. To address potential eosinophilic damage, the patient received empiric treatment for presumptive parasitic infections (praziquantel and albendazole), as well as a brief course of steroid therapy. Interestingly, the AEC began to improve even before the initiation of these therapies, and the ultimate infectious workup for parasitic infections was unremarkable as outlined in Table 1. The patient had a long-standing (for around 10 years) history of HE, with eosinophil percentage ranging from 10% to 18% with the highest absolute amount recorded during hospitalization related to thromboembolic complications. It is worth mentioning that the immunoglobulin E levels were also elevated, but further investigations including myeloproliferative neoplasm (MPN) fluorescence in situ hybridization (FISH), flow cytometry, and Janus kinase 2 (JAK2) V617F cascade were negative. Furthermore, the CMR demonstrated normal left and right ventricular (RV) function (left ventricle ejection fraction {LVEF} of 60.5% and right ventricle ejection fraction {RVEF} of 53%) without wall motion abnormality or MR. T2 images did not reveal any signs of inflammation or edema. No evidence was found for an infiltrative process or late gadolinium enhancement (LGE) to support the diagnosis of eosinophilic cardiac involvement. Importantly, the CMR confirmed the presence of the two LV thrombi on long T1 images, located on the apex and inferolateral walls without evidence of neoplastic masses. Consequently, the patient's anticoagulant therapy was switched to warfarin.

**Table 1:** Relevant workup from a current and prior hospitalization For the PNH panel, the antibodies tested were FLAER, CD59, CD14, CD15, CD24, CD33, glycophorin, and CD45 hs, high-sensitivity; NT-proBNP, N-terminal pro-B-type natriuretic peptide; dsDNA, double-stranded deoxyribonucleic acid; 5-HIAA, 5-hydroxyindoleacetic acid; ANA, antinuclear antibody; ANCA, antineutrophil cytoplasmic antibody; DRVVT, diluted Russell viper venom time; PNH, paroxysmal nocturnal hemoglobinuria; MPN, myeloproliferative neoplasms; TB, tuberculosis; BAL, bronchoalveolar lavage; AFB, acid-fast bacilli; Ig, immunoglobulin; RNP, ribonucleoprotein; DOAC, direct oral anticoagulants; FISH, fluorescence in situ hybridization; JAK2, Janus kinase 2; Ag, antigen; N/A, not available; PDGFRA, platelet-derived growth factor receptor alpha; PDGFRB, platelet-derived growth factor receptor beta; FGFR1, fibroblast growth factor receptor 1; FLAER, fluorescently labeled aerolysin; CD, cluster of differentiation

Laboratory parameter	Results at current admission	Results nine months ago	Reference range
hs Troponin I	267 ng/L	N/A	<35.0 ng/L
NT-proBNP	8,141 pg/mL	1,446 pg/mL	<900 pg/mL
Eosinophils, absolute automated	0.95 × 10³/μL	2.47 × 10³/μL	0.00-0.44 × 10³/μL
Hemoglobin A1C	6.3%	N/A	4.6%-5.6%
Iron	24 µg/dL	N/A	41-168 µg/dL
Transferrin saturation	7%	N/A	15%-50%
5-HIAA	5.7 mg/24 hours	N/A	≤6.0 mg/24 hours
ANA screen	Negative	N/A	Negative
ANCA screen	Negative	Negative	Negative
dsDNA antibody (Ab) screen, serum	Antibodies not present	N/A	Antibodies not present
Anti-Smith (Sm) IgG, serum	3.7	N/A	<20.0 arbitrary units
Anti-RNP IgG, serum	<3.5	N/A	<20.0 arbitrary units
Angiotensin-converting enzyme	51 U/L	N/A	9-67 U/L
Rheumatoid factor, serum	7	N/A	≤12 IU/mL
Cyclic citrullinated peptide Ab, S	16 units	N/A	<20 units
Myeloperoxidase Ab	<1.0 AI	<1.0 AI	<1.0 AI
Proteinase 3 antibody	<1.0 AI	<1.0 AI	<1.0 AI
C3 complement	83 mg/dL	125 mg/dL	82-185 mg/dL
C4 complement	11 mg/dL	19.0 mg/dL	15.0-53.0 mg/dL
Immunoglobulin E	139 kU/L	145 kU/L	≤114 kU/L
Cryoglobulin	N/A	Negative	Negative
Aldolase	6.4 U/L	N/A	≤8.1 U/L
Beta-2-glycoprotein 1 antibody, IgG	N/A	<9.4 U/mL	<15.0 U/mL
Beta-2-glycoprotein 1 antibody, IgM	N/A	<9.4 U/mL	<15.0 U/mL
Lupus anticoagulant (DRVVT screen)	N/A	31 seconds	≤45 seconds
DRVVT: confirm ratio, post DOAC	1.2	N/A	1.0-1.2
Cardiolipin IgG antibody	N/A	<2.0 GPL U/mL	<20.0 GPL U/mL
Cardiolipin IgM antibody	N/A	<2.0 MPL U/mL	<20.0 MPL U/mL
PNH panel	No PNH clones were detected in red or white blood cells	N/A	Negative
Flow cytometry (peripheral blood)	No abnormalities identified	No abnormalities identified	Negative
MPN FISH panel	Negative PDGFRA, PDGFRB, and FGFR1 rearrangement	N/A	Negative
JAK2 V617F cascade	Negative	N/A	Negative
Syphilis screen IgG and IgM	N/A	Nonreactive	Nonreactive
*Trypanosoma cruzi* Ab, IgG, S	N/A	Negative	Negative
*Strongyloides* Ab, IgG	Negative	Negative	Negative
*Toxocara* antibody IgG	N/A	Negative	Negative
Cysticercosis, IgG Ab, western blot	N/A	Negative	Negative
Stool ova and parasites	N/A	Negative	Negative
T-SPOT, TB test	N/A	Negative	Negative
QuantiFERON Gold	N/A	Negative	Negative
BAL AFB stain and culture	N/A	Negative	Negative
BAL fungal culture and stain	N/A	Negative	Negative
HIV-1/2 Ag/Ab	Nonreactive	N/A	Nonreactive

The patient's clinical course remained uneventful until approximately one month before the current presentation, at which point he presented with dyspnea and PND and was hospitalized with the diagnosis of congestive heart failure for a brief IV diuretics.

The home medications included empagliflozin, spironolactone, metoprolol succinate, warfarin, aspirin, atorvastatin, flecainide, metformin, bumetanide, and levothyroxine. There was no pertinent cardiovascular family history, as well as a history of tobacco use, alcohol consumption, or drug abuse.

The initial vital signs were as follows: a temperature of 36.8°C, a heart rate of 101 beats per minute, a blood pressure of 121/79 mmHg, a respiratory rate of 16 breaths per minute, and an oxygen saturation of 97% on room air. A physical examination revealed cachexia, with an otherwise regular rate and rhythm, a normal S1 and S2, and an absence of S3, S4, murmurs, rubs, gallops, jugular venous distention, or peripheral edema. Pulmonary auscultation was negative for rales, wheezing, or rhonchi. The initial workup was notable for an elevated N-terminal pro-B-type natriuretic peptide (NT-proBNP) and minimally elevated "flat" troponin levels. The relevant workup for the current and prior admission (nine months ago) is outlined in Table 1, including hypercoagulability, rheumatology, malignancy, and infectious domains, all of which were nonrevealing.

**Figure 2:**
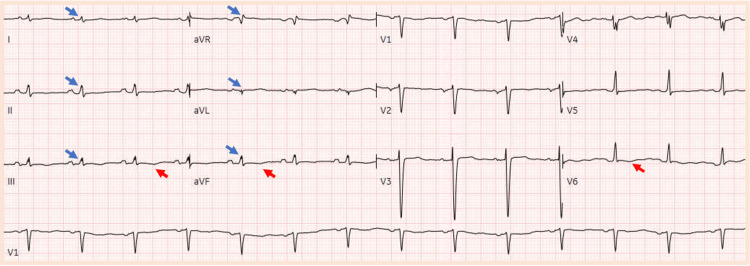
The ECG from the current hospitalization The blue arrows indicate the presence of low voltage in the limb leads, and the red arrows indicate T-wave inversions in III, aVF, and V6 leads.

The ECG demonstrated low voltage in the limb leads and small T-wave inversions in leads III, aVF, and V6. No other significant abnormalities were noted as depicted in Figure 2.

**Figure 3:**
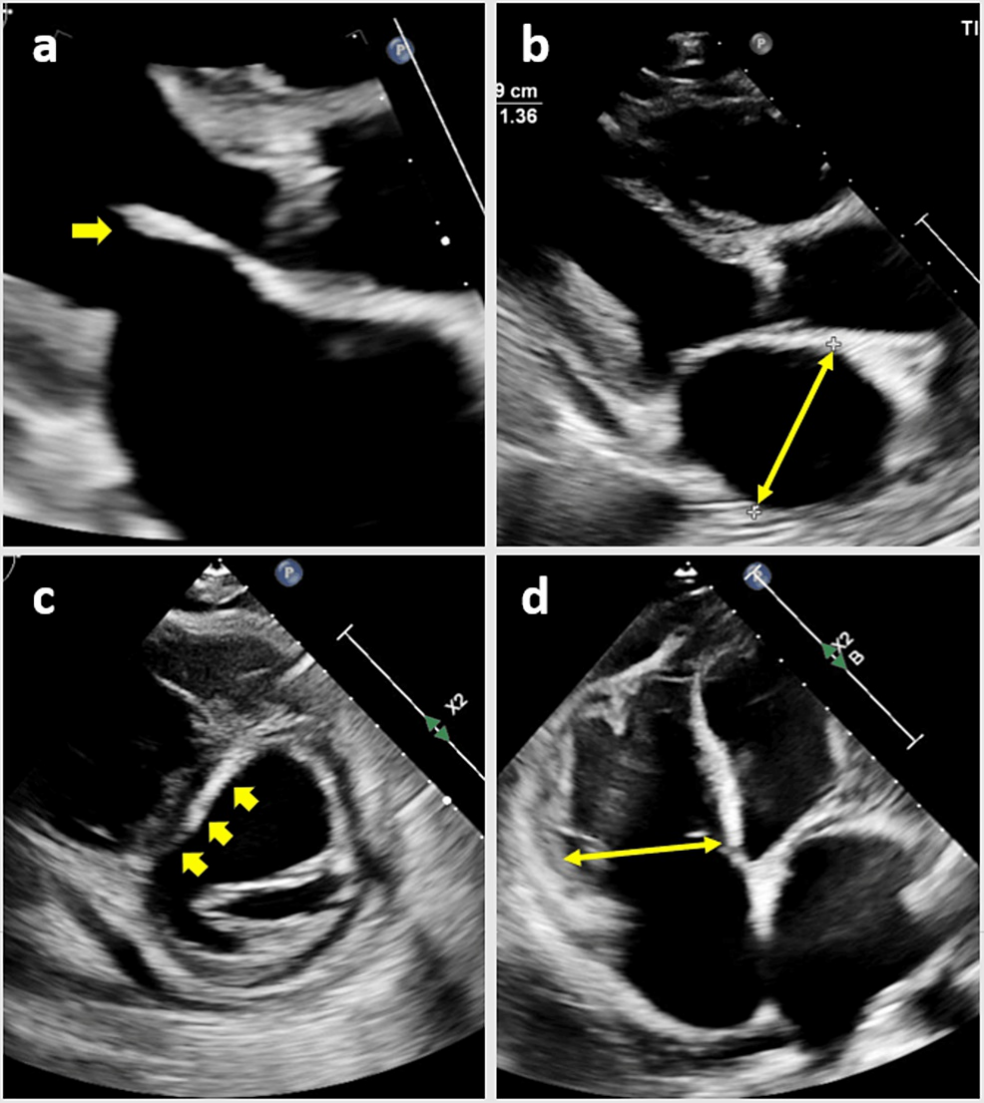
The TTE from the current hospitalization (a) Mildly thickened anterior mitral valve leaflet. (b) Severe left atrial dilation. (c and d) Interventricular septal flattening with a "D" shape configuration and severe right ventricular dilation, respectively TTE: transthoracic echocardiogram

The initial TTE identified normal LV size with an EF of 51%, a restrictive filling pattern, and a severely dilated right ventricle with severely decreased function. Interestingly, the LV wall thickness was normal. Other significant findings included severe PHTN with right ventricular systolic pressure (RVSP) of 80 mmHg. The mitral valve was mildly thickened with severe central MR, postulated to be "atrial" in origin given the severely dilated left atrium. Of note, there were moderate tricuspid regurgitation and septal flattening both during systole and diastole. The TTE findings are depicted in Figure 3.

It is important to note that the subsequent transesophageal echocardiogram confirmed a structurally normal mitral valve with functional moderate MR.

Despite unremarkable chest X-ray, the computed tomography (CT) angiography of the chest, conducted on the next day of admission, revealed bilateral small pleural effusions with signs of pulmonary edema but was negative for pulmonary embolism. Subsequently, a CT scan of the chest, abdomen, and pelvis performed as part of a weight loss workup ruled out any neoplastic processes.

For the evaluation of cardiac infiltrative diseases, the patient underwent a CMR that was again negative for the infiltrative process or eosinophilic cardiac involvement but revealed severe RV dysfunction (RVEF: 24%), moderate MR, and small (5 × 7mm) LV apical thrombus. The LVEF was 52%.

The right heart catheterization confirmed elevated left-sided filling pressure with severe PHTN and severely decreased cardiac output as presented in Table 2.

**Table 2:** The right heart catheterization results from the current hospitalization

Parameter	Result	Reference range
Right atrial mean pressure	6 mmHg	0-8 mmHg
Pulmonary artery wedge pressure	30 mmHg	6-15 mmHg
Pulmonary artery mean pressure	43 mmHg	10-20 mmHg
Fick: cardiac output	1.78 L/minute	5-6 L/minute
Fick: cardiac index	1.17 L/minute/m²	2.8-4.2 L/minute/m²
Pulmonary vascular resistance	5.06 WU	1.9-3.1 WU
Systemic vascular resistance	3,191 dyn × second/cm⁵	900-1,400 dyn × second/cm⁵

Consequently, the patient was started on inotropic therapy to address the cardiogenic shock. The dual-energy computed tomography pulmonary angiogram for the evaluation of the precapillary component of PHTN was negative for chronic thromboembolic pulmonary hypertension (CTEPH). The cardiogenic shock was postulated to be secondary to severe right heart failure due to restrictive cardiomyopathy causing severe PHTN. Eventually, the EMB was negative for myocarditis, eosinophils, amyloid deposition, or an iron stain, and the left heart catheterization identified nonobstructive coronary artery disease (CAD). As a result of this comprehensive workup, the patient was diagnosed with left-sided restrictive cardiomyopathy with LVT and RV failure potentially attributed to HES. The patient was discharged on home milrinone with the plan of reevaluation for heart transplantation after the improvement of cachexia.

## Discussion

The patient had a history of LVT and associated thromboembolic complications starting nine months prior to the current presentation. Several potential causes of LVT, such as ischemic etiology, systolic dysfunction, cardiac amyloidosis, LV noncompaction, hypertrophic cardiomyopathy (HCM), autoimmune disorders, and malignancy, were deemed to be unlikely given the normal EF, nonobstructive CAD, lack of evidence of noncompaction or HCM on TTE and CMR, absence of amyloidosis on biopsy, normal autoimmune markers, and negative imaging studies for malignancies. Furthermore, the patient was not on any hormonal or other medications predisposing to thromboembolic complications. Importantly, potential causes of increased coagulability causing arterial thrombosis, such as antiphospholipid syndrome (APS) and paroxysmal nocturnal hemoglobinuria panels, showed no alterations. Collectively, all these data indicate that the most likely underlying cause of LVT was HES, although the thrombi were free-floating and not obliterating the LV apex as typically seen in cases of EM. An additional distinctive feature of this case was the presence of LVT in the absence of conventional eosinophilic cardiac involvement, typically observed as EM or Loffler endomyocarditis, since both the initial CMR performed when the patient was found to have two large intraventricular thrombi, along with the highest AEC, and the subsequent CMR, performed during current hospitalization, were not supportive of these entities. Additionally, the EMB performed nine months after the inciting event failed to provide conclusive evidence of eosinophilic cardiac involvement. It is essential to acknowledge that the biopsy might miss the diagnosis of HES given the possibility of patchy eosinophilic involvement of the endomyocardium, as well as a relatively improved eosinophil count at the time of biopsy.

In the evaluation of the potential causes of HE, the infectious workup, including stool ova and parasites, parasitic serology, fungal studies, HIV, and syphilis, was nonrevealing. However, the patient received a course of empiric antiparasitic treatment and a short course of steroids, although the eosinophil count decreased unexpectedly even before these treatments. Furthermore, the evaluation for antinuclear antibodies (ANA), antineutrophil cytoplasmic antibodies (ANCA), double-stranded deoxyribonucleic acid (dsDNA) antibody screen, myeloperoxidase (MPO) antibodies (Ab), and proteinase 3 (PR3) antibodies returned negative. Additionally, the clinical presentation was not consistent with the connective-tissue-disease-related etiologies of HE. It is worth mentioning that extensive imaging studies for malignancies were negative, ruling out another potential cause of secondary HE. The investigations for the primary causes of HE, including the myeloproliferative neoplasm (MPN) FISH panel, JAK2 V617F cascade, and flow cytometry, were also noncontributory. The elevated immunoglobulin E level is nonspecific and can be observed both in primary and in secondary HE, as well as in HES. As a result of this extensive workup, HES was considered to be the most likely underlying cause of HE.

We could identify two cases meeting our search criteria as shown in Table 3 [4,5].

**Table 3:** Characteristics of the identified cases HES, hypereosinophilic syndrome; HE, hypereosinophilia; CMR, cardiac magnetic resonance imaging; EMB, endomyocardial biopsy; LMWH, low-molecular-weight heparin

Author	Year	Journal	Age (years)	Sex	Type of HE	Eosinophil account	Workup	Treatment
Klion [4]	2015	Blood	42	Female	HES	8.0 × 10⁹/L	CMR and EMB	Methylprednisolone, LMWH imatinib, alfa interferon, and mepolizumab
Zou et al. [5]	2022	Front Pharmacol	44	Female	Idiopathic HES	2.06 × 10⁹/L	CMR	Prednisone, rivaroxaban, and aspirin
Our case	2024	Cureus	63	Male	HES	2.47 × 10⁹/L	CMR and EMB	Methylprednisolone, warfarin, and antiparasitic

History, epidemiology, and terminology

The term HES was first described by Hardy and Anderson in 1968. It assumes that absolute HE causes end-organ damage due to eosinophilic infiltration [6]. Later, Chusid et al. defined the characteristic features of HES, including AEC of >1,500/μL persisting for longer than six months, with evidence of end-organ involvement, and the exclusion of secondary causes of HES [7]. Nowadays, the persistence of HE for longer than six months is not a universally accepted criterion for HES, considering the advancements in diagnostic technology and the ability to detect primary and secondary causes of HE earlier, as well as the need for urgent therapy in certain cases to prevent organ damage. The most common age range for the diagnosis of HES is typically from 20 to 50 years, though there are some data about diagnosing it in the pediatric population [8]. The most commonly affected organ systems are the skin, lungs, and gastrointestinal and, less commonly, cardiovascular systems [9]. The cardiovascular system has been reported to be involved in around 50% of cases [10]. It is important to note that regardless of the etiology of HE, it may cause serious life-threatening cardiovascular complications [10]. While HE is mandatory for the diagnosis of EM, it should be noted that there are reports of eosinophilic cardiac involvement in the absence of peripheral eosinophilia [11-13]. The two well-described manifestations of cardiac involvement in HES are EM and its chronic form known as Loeffler endocarditis. The latter is the sequelae of EM, presenting as restrictive cardiomyopathy due to fibrotic changes, and was first described by William Loeffler in 1936. The terms such as Loeffler endocarditis, eosinophilic endomyocardial disease, and fibroblastic endocarditis are used interchangeably and are considered historical terms to refer to the fibrotic stage of EM. Other manifestations of hypereosinophilic cardiac damage include dilated cardiomyopathy and constrictive pericarditis [14-16]. For the referral of hypereosinophilic cardiac damage, we will use the collective term EM in this review. We believe that all the manifestations of this entity are the sequelae of eosinophil-induced inflammation.

Pathophysiology

Cardiac involvement in HES has been proposed to occur in three distinct stages: necrotic, thrombotic, and fibrotic. In the initial necrotic stage, eosinophils infiltrate cardiac tissue and release cationic proteins, leading to myocardial necrosis and microabscess formation. This is followed by the thrombotic stage, characterized by thrombus formation on the damaged endocardium. Potential mechanisms contributing to thrombogenesis include increased tissue factor expression by eosinophils, along with the circulating inflammatory, oxidative, and prothrombotic factors such as major basic protein (MBP) and eosinophil peroxidase, contained in eosinophilic granules, predisposing to thrombogenicity [17-20]. It is hypothesized that the principal product of eosinophil peroxidase, called hypothiocyanous acid (HOSCN), may serve as another potent tissue factor inducer, further exacerbating thrombogenicity [21]. Moreover, some data suggest that molecular abnormalities may confer a fivefold increased risk of thrombogenicity in individuals with HES [2]. The final fibrotic stage causes altered cardiac function and heart failure as a manifestation of restrictive cardiomyopathy (known as Loeffler endocarditis) or fibrosis of cardiac leaflets leading to mitral or tricuspid regurgitation [22]. Interestingly, EM may involve the LV, RV, or both ventricles [23,24]. The proposed underlying mechanisms of fibrosis in EM include transforming growth factor-alpha (TGF-α) and eosinophil-derived neurotoxin-induced fibroblast proliferation, as well as extracellular matrix deposition mediated by transforming growth factor-beta (TGF-β) [25-27].

Clinical presentation

The first stage, characterized by eosinophilic cardiac infiltration and necrosis, is largely clinically silent. In the second stage, thromboembolic complications may occur, presenting a poor prognosis in EM. It complicates 33% of Loeffler endocarditis cases, with a mortality rate of 27% [28]. While dyspnea is the most common manifestation, fatigue, cough, arrhythmia, and thrombotic complications may also occur in the fibrotic stage characterized by restrictive heart failure [23,29]. Patients may also present with fever and rash, and weight loss may further complicate end-stage heart failure [30,31].

Diagnosis

There are no pathognomonic ECG findings for EM. However, a commonly observed ECG finding is T-wave inversion.

TTE in the thrombotic or fibrotic stages of EM may detect intracavitary thrombi. In the stage of fibrotic changes, TTE may reveal a restrictive filling pattern, as well as endomyocardial thickening, obliterating apical thrombus, and posterior mitral valve involvement with MR [32,33]. The thrombotic process may extend from the apex to the subvalvular regions, leading to the complete obliteration of the ventricle. The identification of left or right ventricular apical obliterating thrombus in conjunction with preserved ejection fraction and peripheral eosinophilia strongly suggests EM. Although aortic or pulmonary valve involvement in EM is rare, atrioventricular valve involvement is common, leading to the development of valvular regurgitation through fibrotic or thrombotic subvalvular apparatus involvement [34,35]. Frequent thrombotic and fibrotic changes occurring in the posterior LV wall can lead to the entrapment of the chordae tendineae, affecting the posterior leaflet of the mitral valve and leading to eccentric MR [22]. Cases of both native and prosthetic mitral valve thrombosis have been described [36,37].

In the necrotic stage of myocarditis, CMR may demonstrate increased T2 signal and LGE, indicating edema and inflammation. Subsequently, during the thrombotic stage, CMR can identify thrombi in the ventricular apices [33]. LGE during the chronic fibrotic stage of the disease is consistent with fibrosis [38]. Interestingly, LGE may exhibit a patchy or diffuse distribution extending from the apex to the subvalvular regions. A pathognomonic CMR finding of fibrosis is the "V sign" localized in the apex during delayed enhancement, characterized by a three-layered appearance of dark overlying thrombus, bright fibrotic endomyocardium, and dark underlying myocardium [39,40]. CMR also plays an invaluable role in detecting intracavitary thrombi, offering higher sensitivity and specificity compared to TTE. In certain cases, apical thrombus can be easily mistaken for apical hypertrophy, making CMR essential for accurate diagnosis [28]. The absence of delayed enhancement in the thrombotic mass helps to differentiate it from surrounding cardiac tissues, given the avascular nature of the thrombus and the lack of contrast uptake [40]. It is important to note that the stages of EM may overlap, leading to imaging findings that detect characteristic features of different stages simultaneously. CMR has crucial diagnostic significance, being useful in every stage of EM development [38,41,42].

Though echocardiography and CMR are considered the most commonly used imaging modalities in EM, cardiac CT scan and 18F-fluorodeoxyglucose (18F-FDG) positron emission tomography (PET) are two alternatives for certain patients. Cardiac CT can be considered in cases of a poor acoustic window or when patients have contraindications to CMR. Nevertheless, radiation exposure, the risk of contrast-related complications, limited soft tissue resolution, and the lack of functional assessment of LV hemodynamics minimize the utility of cardiac CT as a preferred diagnostic modality for EM. 18F-FDG PET may detect active inflammation and guide therapy at early stages of EM, as well as reveal irreversible fibrosis when CMR is inconclusive or contraindicated. Since there is no good evidence for the utility of cardiac CT and 18F-FDG PET scan in EM, these modalities should be considered as second-line imaging tools for the detection of EM in selected patients.

The EMB is still considered the gold standard for diagnosing EM but should be reserved for cases when CMR is nondiagnostic [43]. During the initial necrotic stage, biopsy may detect interstitial eosinophilic infiltration and inflammation with myocyte necrosis and edema. In the late stages, endomyocardial fibrosis may predominate without inflammatory infiltrates. However, due to the patchy nature of myocardial involvement, the biopsy may sometimes miss the diagnosis. Interestingly, following treatment, the biopsy may detect the resolution of EM, showing signs of replacement fibrosis [44]. Certain data indicate that the sensitivity of EMB for Loffler endocarditis is around 50% [45]. Given the potential of negative hematoxylin and eosin staining of eosinophils, it is imperative to perform an immunohistologic examination with fluorescein-conjugated antibodies for the detection of eosinophil granule MBP, which plays an important role in thrombogenesis and may aid in the tissue diagnosis of EM [46].

Treatment

The treatment targets in EM should focus on three main aspects: the initial management of hypereosinophilic cardiac involvement, a thorough workup for HE to determine targeted etiologic therapy, and the treatment of diverse cardiovascular manifestations such as heart failure (including surgical interventions) and thrombotic complications.

The literature currently lacks well-validated studies specifically addressing the control of HE in EM. The treatment recommendations discussed below are primarily extrapolated from therapies for HES rather than being tailored specifically for the management of EM. The first-line treatment of HES-induced EM is considered steroid therapy [4]. Prednisone at a dose of 1 mg/kg should be started promptly when life-threatening manifestations are present or imminent. In a retrospective analysis, around 85% of patients achieved either complete or partial response to steroid therapy after one month of treatment [9]. If EM is not responsive to steroids, the second-line choices are hydroxyurea or interferon-α [9,30,37,47]. The workup for the etiologies of HE is strongly encouraged, including exploring secondary causes of HE, as it may help determine the treatment strategy. Following the exclusion of secondary or reactive causes of HE, the diagnostic efforts should focus on blood and marrow evaluation to detect hematolymphoid neoplasms. Particularly, if genetic testing reveals PDGFRA/B mutation, irrespective of cardiac involvement, a tyrosine kinase inhibitor, imatinib, should be initiated [48,49]. It should be noted that the discontinuation or dose reduction of imatinib following complete remission at a daily dose of 300-400 mg results in disease relapse, highlighting the importance of continuous therapy [50]. In recent years, eosinophil-targeted therapy has gained popularity for the treatment of HES. The humanized monoclonal anti-IL-5 antibody, mepolizumab, has been demonstrated to improve outcomes [51-53]. Mepolizumab is currently an FDA-approved agent for managing HES, particularly effective in reducing the frequency of flares and serving as a steroid-sparing therapy [52].

There are no specific therapies for heart failure management in EM. The treatment approach is not different from generally accepted therapies used in heart failure management with underlying restrictive cardiomyopathy. It has been noted that thromboembolic complications are challenging to control in EM, particularly due to the release of eosinophilic granular proteins, which could neutralize thrombomodulin's local anticoagulant activity, resulting in intracavitary thrombosis and thromboembolism [54]. Consistent with this observation, HE leads to a lower rate of thrombus resolution with anticoagulation and predisposes to more frequent thromboembolic complications and increased mortality compared with other causes of LVT. Despite existing controversies stemming from treatment failure, anticoagulation in LVT should still be considered, supported by some low-level evidence indicating favorable outcomes [28]. There are no universally accepted guidelines for anticoagulation. Currently, warfarin is the predominant agent used for thromboembolic complications in HES [23,28]. The experience with direct oral anticoagulants (DOACs) is less common. Though there is an increased risk of thrombogenesis in HES, routine anticoagulation is not recommended unless there is a confirmed intracavitary thrombus or other manifestation of thromboembolism. In patients with a history of thromboembolic complications, preventive anticoagulation may be warranted [55,56].

In the late fibrotic stages of EM, surgical intervention, particularly endocardial decortication, and atrioventricular valve replacement or repair are frequently employed practices that improve outcomes [57,58].

## Conclusions

HE, irrespective of its underlying etiology, should be recognized as a high-risk thrombotic condition predisposing to the development of LVT including in patients with preserved ejection fraction. Cardiac involvement in HES is not uncommon and warrants consideration in cases of peripheral HE, restrictive cardiomyopathy, and LVT even when CMR and EMB do not support the diagnosis of EM. Given the patchy nature of the process and the decreasing probability of detecting eosinophils in the late fibrotic stages, the EMB may fail to diagnose EM accurately. When clinical suspicion for EM is high despite inconclusive EMB and CMR findings, clinicians should consider secondary diagnostic modalities such as 18F-FDG PET, which can enhance diagnostic accuracy. The early initiation of steroid therapy in EM is essential to prevent myocardial damage while awaiting etiologic workup for the underlying cause of HE. Although definitive evidence is still lacking, existing data support the use of warfarin for anticoagulation.

## References

[1] Shomali W, Gotlib J (2022). World Health Organization-defined eosinophilic disorders: 2022 update on diagnosis, risk stratification, and management. Am J Hematol.

[2] Leiva O, Baker O, Jenkins A (2021). Association of thrombosis with hypereosinophilic syndrome in patients with genetic alterations. JAMA Netw Open.

[3] Wallace KL, Elias MK, Butterfield JH, Weiler CR (2013). Hypereosinophilic syndrome and thrombosis: a retrospective review. J Allergy Clin Immunol.

[4] Klion AD (2015). How I treat hypereosinophilic syndromes. Blood.

[5] Zou M, Liu G, Li Y (2022). Case report: application of non-VKA oral anticoagulants in patient of idiopathic hypereosinophilic syndrome with intracardiac thrombus. Front Pharmacol.

[6] Hardy WR, Anderson RE (1968). The hypereosinophilic syndromes. Ann Intern Med.

[7] Chusid MJ, Dale DC, West BC, Wolff SM (1975). The hypereosinophilic syndrome: analysis of fourteen cases with review of the literature. Medicine (Baltimore).

[8] Crane MM, Chang CM, Kobayashi MG, Weller PF (2010). Incidence of myeloproliferative hypereosinophilic syndrome in the United States and an estimate of all hypereosinophilic syndrome incidence. J Allergy Clin Immunol.

[9] Ogbogu PU, Bochner BS, Butterfield JH (2009). Hypereosinophilic syndrome: a multicenter, retrospective analysis of clinical characteristics and response to therapy. J Allergy Clin Immunol.

[10] Weller PF, Bubley GJ (1994). The idiopathic hypereosinophilic syndrome. Blood.

[11] Fozing T, Zouri N, Tost A, Breit R, Seeck G, Koch C, Oezbek C (2014). Management of a patient with eosinophilic myocarditis and normal peripheral eosinophil count: case report and literature review. Circ Heart Fail.

[12] Brambatti M, Matassini MV, Adler ED, Klingel K, Camici PG, Ammirati E (2017). Eosinophilic myocarditis: characteristics, treatment, and outcomes. J Am Coll Cardiol.

[13] Hagendorff A, Hümmelgen M, Omran H (1998). [Löffler fibroblastic endocarditis in the thrombotic stages in isolated right ventricular tissue eosinophilia] (Article in German). Z Kardiol.

[14] DePace NL, Nestico PF, Morganroth J (1983). Dilated cardiomyopathy in the idiopathic hypereosinophilic syndrome. Am J Cardiol.

[15] Christen R, Morant R, Schneider J, Jenni R, Fehr J (1989). Progressive dilated cardiomyopathy in a patient with longstanding and complete prednisone-induced hematological remission of idiopathic hypereosinophilic syndrome. Klin Wochenschr.

[16] Lui CY, Makoui C (1988). Severe constrictive pericarditis as an unsuspected cause of death in a patient with idiopathic hypereosinophilic syndrome and restrictive cardiomyopathy. Clin Cardiol.

[17] Cugno M, Marzano AV, Lorini M, Carbonelli V, Tedeschi A (2014). Enhanced tissue factor expression by blood eosinophils from patients with hypereosinophilia: a possible link with thrombosis. PLoS One.

[18] Moosbauer C, Morgenstern E, Cuvelier SL (2007). Eosinophils are a major intravascular location for tissue factor storage and exposure. Blood.

[19] Slungaard A, Vercellotti GM, Tran T, Gleich GJ, Key NS (1993). Eosinophil cationic granule proteins impair thrombomodulin function. A potential mechanism for thromboembolism in hypereosinophilic heart disease. J Clin Invest.

[20] Rohrbach MS, Wheatley CL, Slifman NR, Gleich GJ (1990). Activation of platelets by eosinophil granule proteins. J Exp Med.

[21] Wang JG, Mahmud SA, Thompson JA, Geng JG, Key NS, Slungaard A (2006). The principal eosinophil peroxidase product, HOSCN, is a uniquely potent phagocyte oxidant inducer of endothelial cell tissue factor activity: a potential mechanism for thrombosis in eosinophilic inflammatory states. Blood.

[22] Gottdiener JS, Maron BJ, Schooley RT, Harley JB, Roberts WC, Fauci AS (1983). Two-dimensional echocardiographic assessment of the idiopathic hypereosinophilic syndrome. Anatomic basis of mitral regurgitation and peripheral embolization. Circulation.

[23] Kariyanna PT, Hossain NA, Onkaramurthy NJ, Jayarangaiah A, Hossain NA, Jayarangaiah A, McFarlane IM (2021). Hypereosinophilia and Löffler’s endocarditis: a systematic review. Am J Med Case Rep.

[24] Alam A, Thampi S, Saba SG, Jermyn R (2017). Loeffler endocarditis: a unique presentation of right-sided heart failure due to eosinophil-induced endomyocardial fibrosis. Clin Med Insights Case Rep.

[25] Noguchi H, Kephart GM, Colby TV, Gleich GJ (1992). Tissue eosinophilia and eosinophil degranulation in syndromes associated with fibrosis. Am J Pathol.

[26] Lyons RM, Moses HL (1990). Transforming growth factors and the regulation of cell proliferation. Eur J Biochem.

[27] Barnard JA, Lyons RM, Moses HL (1990). The cell biology of transforming growth factor beta. Biochim Biophys Acta.

[28] Zhang Q, Si D, Zhang Z, Zhang W (2021). Loeffler endocarditis with intracardiac thrombus: case report and literature review. BMC Cardiovasc Disord.

[29] Parrillo JE, Borer JS, Henry WL, Wolff SM, Fauci AS Fauci AS (1997). The cardiovascular manifestations of the hypereosinophilic syndrome. Prospective study of 26 patients, with review of the literature. Am J Med.

[30] Fauci AS, Harley JB, Roberts WC, Ferrans VJ, Gralnick HR, Bjornson BH (1982). NIH conference. The idiopathic hypereosinophilic syndrome. Clinical, pathophysiologic, and therapeutic considerations. Ann Intern Med.

[31] Spry CJ (1982). The hypereosinophilic syndrome: clinical features, laboratory findings and treatment. Allergy.

[32] Mankad R, Bonnichsen C, Mankad S (2016). Hypereosinophilic syndrome: cardiac diagnosis and management. Heart.

[33] Polito MV, Hagendorff A, Citro R (2020). Loeffler’s endocarditis: an integrated multimodality approach. J Am Soc Echocardiogr.

[34] Bozcali E, Aliyev F, Agac MT, Erkan H, Okcun B, Babalik E, Karpuz H (2007). Unusual case of aortic valve involvement in patient with Löffler's endomyocarditis: management, follow-up and short review of the literature. J Thromb Thrombolysis.

[35] Garg A, Nanda NC, Sungur A, Sharma GL, Mehta KJ, Öz TK (2014). Transthoracic echocardiographic detection of pulmonary valve involvement in Löeffler's endocarditis. Echocardiography.

[36] Zakhama L, Slama I, Boussabah E (2014). Recurrent native and prosthetic mitral valve thrombosis in idiopathic hypereosinophilic syndrome. J Heart Valve Dis.

[37] Watanabe K, Tournilhac O, Camilleri LF (2002). Recurrent thrombosis of prosthetic mitral valve in idiopathic hypereosinophilic syndrome. J Heart Valve Dis.

[38] Kleinfeldt T, Ince H, Nienaber CA (2011). Hypereosinophilic syndrome: a rare case of Loeffler's endocarditis documented in cardiac MRI. Int J Cardiol.

[39] Jariwala N, McGraw S, Rangarajan VS, Mirza O, Wong J, Farzaneh-Far A (2015). The 'V' sign of endomyocardial fibrosis. QJM.

[40] Salemi VM, Rochitte CE, Shiozaki AA (2011). Late gadolinium enhancement magnetic resonance imaging in the diagnosis and prognosis of endomyocardial fibrosis patients. Circ Cardiovasc Imaging.

[41] Debl K, Djavidani B, Buchner S (2008). Time course of eosinophilic myocarditis visualized by CMR. J Cardiovasc Magn Reson.

[42] Syed IS, Martinez MW, Feng DL, Glockner JF (2008). Cardiac magnetic resonance imaging of eosinophilic endomyocardial disease. Int J Cardiol.

[43] Dominguez F, Kühl U, Pieske B, Garcia-Pavia P, Tschöpe C (2016). Update on myocarditis and inflammatory cardiomyopathy: reemergence of endomyocardial biopsy. Rev Esp Cardiol (Engl Ed).

[44] Getz MA, Subramanian R, Logemann T, Ballantyne F (1991). Acute necrotizing eosinophilic myocarditis as a manifestation of severe hypersensitivity myocarditis. Antemortem diagnosis and successful treatment. Ann Intern Med.

[45] Allderdice C, Marcu C, Kabirdas D (2018). Intracardiac thrombus in leukemia: role of cardiac magnetic resonance imaging in eosinophilic myocarditis. CASE (Phila).

[46] Wright BL, Leiferman KM, Gleich GJ (2011). Eosinophil granule protein localization in eosinophilic endomyocardial disease. N Engl J Med.

[47] Butterfield JH, Gleich GJ (1994). Response of six patients with idiopathic hypereosinophilic syndrome to interferon alfa. J Allergy Clin Immunol.

[48] Klion AD, Robyn J, Akin C (2004). Molecular remission and reversal of myelofibrosis in response to imatinib mesylate treatment in patients with the myeloproliferative variant of hypereosinophilic syndrome. Blood.

[49] Helbig G, Stella-Hołowiecka B, Majewski M (2008). A single weekly dose of imatinib is sufficient to induce and maintain remission of chronic eosinophilic leukaemia in FIP1L1-PDGFRA-expressing patients. Br J Haematol.

[50] Klion AD, Robyn J, Maric I (2007). Relapse following discontinuation of imatinib mesylate therapy for FIP1L1/PDGFRA-positive chronic eosinophilic leukemia: implications for optimal dosing. Blood.

[51] Wechsler ME, Fulkerson PC, Bochner BS (2012). Novel targeted therapies for eosinophilic disorders. J Allergy Clin Immunol.

[52] Roufosse FE, Kahn JE, Gleich GJ (2013). Long-term safety of mepolizumab for the treatment of hypereosinophilic syndromes. J Allergy Clin Immunol.

[53] Rothenberg ME, Klion AD, Roufosse FE (2008). Treatment of patients with the hypereosinophilic syndrome with mepolizumab. N Engl J Med.

[54] Mukai HY, Ninomiya H, Ohtani K, Nagasawa T, Abe T (1995). Major basic protein binding to thrombomodulin potentially contributes to the thrombosis in patients with eosinophilia. Br J Haematol.

[55] Ono R, Iwahana T, Kato H, Okada S, Kobayashi Y (2021). Literature reviews of stroke with hypereosinophilic syndrome. Int J Cardiol Heart Vasc.

[56] Levine GN, McEvoy JW, Fang JC (2022). Management of patients at risk for and with left ventricular thrombus: a scientific statement from the American Heart Association. Circulation.

[57] Moraes F, Lapa C, Hazin S, Tenorio E, Gomes C, Moraes CR (1999). Surgery for endomyocardial fibrosis revisited. Eur J Cardiothorac Surg.

[58] Harley JB, McIntosh CL, Kirklin JJ, Maron BJ, Gottdiener J, Roberts WC, Fauci AS (1982). Atrioventricular valve replacement in the idiopathic hypereosinophilic syndrome. Am J Med.

